# Effect of Fiber Surface Modification on the Interfacial Adhesion and Thermo-Mechanical Performance of Unidirectional Epoxy-Based Composites Reinforced with Bamboo Fibers

**DOI:** 10.3390/molecules24152682

**Published:** 2019-07-24

**Authors:** Fang Wang, Min Lu, Shujue Zhou, Zhisong Lu, Siyan Ran

**Affiliations:** 1 School of Materials and Energy, Southwest University, Chongqing 400715, China; 2 School of Mathematics and Statistics, Southwest University, Chongqing 400715, China

**Keywords:** natural fiber composites, alkali treatment, interfacial bonding, thermal performance, mechanical properties

## Abstract

In this work, bamboo fibers are chemically modified with NaOH solution of 1, 4, and 7 wt% concentrations at room temperature, respectively, and subsequently the untreated and treated fibers are prepared with epoxy resin for unidirectional composites by hot pressing molding technique. Tensile and micro-bond tests are conducted on the composite specimens to obtain mechanical properties, such as tensile strength and modulus, elongation at break, and interfacial strength. Besides, scanning electron microscopy (SEM) is employed to perform morphological observations for constituent damages. In addition, the influence of alkali concentration on the thermal performance of epoxy-based composites is examined by using differential scanning calorimetry (DSC) and thermogravimetric (TG) analysis. It is found that composite tensile strength reaches the maximum when the alkali concentration is 4%, increased by 45.24% compared with untreated composites. The composite elongation at break increases on increasing the concentration. Inversely, the composite modulus decreases as the concentration increases. Besides, the results demonstrate that the chemical treatment on the fiber surface could improve interface adhesion, as observed from its topography by SEM. Micro-bond test reveals that there is maximum interfacial shear strength when the alkali concentration is 4%, which increases by 100.30% in comparison with the untreated samples. In case of thermal properties, the DSC analysis indicates that the glass transition temperature is maximized at 4% alkali concentration, which is increased by 12.95%, compared to those from unmodified fibers. In addition, TG results show that the 4% concentration also facilitates thermal stability improvement, indicative of superior interfacial bonding.

## 1. Introduction

In recent years, natural fiber composites as a class of environmentally friendly materials possess excellent mechanical properties being in favor of engineering plastics, and have attracted increasing attention in the fields of construction, transportation, aerospace, and new energy vehicles [1]. For example, such composites have replaced traditional wood in terms of furniture products. As a bast plant, bamboo fibers are usually fetched from the bamboo stems after extracting the vascular bundle sheath by several physical methods [2], exhibiting a statistical fracture behavior [3,4]. Among the well-known natural fibers, bamboo fibers have many extraordinary mechanical properties due to inherent properties [2], and thus they provide preferable tensile strength and flexural strength for the resultant composites [5,6]. Epoxy resin, as one of the most commonly-used thermosets, is usually employed as a matrix and adhesive for preparing composites. In view of high modulus and high temperature performance, the epoxy has been widely used in various engineering applications. However, there is no denying that epoxy resin has many disadvantages, such as distinct brittleness due to its cross-linked network structure [7,8]. This is one of the reasons that such composites have not completely replaced conventional composite materials in high-load applications.

More importantly, natural fiber composites always exhibit weak interfacial compatibility between these combined constituents, which is explained by the fact that hydroxyl groups on the fiber surface absorb water molecules and form hydrogen bonds, impeding an intimate intermolecular contact with a hydrophobic polymer matrix [9,10]. As a result, bamboo fibers cannot be easily penetrated into the matrix during the process of composite fabrication, resulting in fiber non-uniform dispersion within the matrix [11,12]. The poor interaction between fibers with matrix reduces the composite interfacial compatibility, leading to ineffective load transfer from the matrix to neighboring fibers. Ultimately, it produces a negative effect on composite mechanical properties [13]. Hence, how to improve interfacial adhesion becomes one of critical issues that promote a wider development of such bio-composites. Kim et al. [14] found that the water absorption of bamboo fibers was degraded after chemical modification, due to the effective removal of hemicellulose and lignin from the fiber surface, indicating that its hydrophilicity was weakened. Wang et al. [15,16] studied the influence of alkali treatment on the morphological and thermomechanical properties of bamboo fibers, and reported that the appearance of fibrillary structure after treatment caused an increase in the contact area available for superior bonding with the matrix, resulting in enhanced interfacial wettability. In addition, Wang et al. [17] conducted an experimental investigation on the effect of alkali treatment of bamboo fibers on the PLA-based composite properties, indicating that the fiber surface treatment could allow for stronger mechanical interlocking effect with the matrix, which was beneficial for superior interfacial performance.

It is justified that the fiber/matrix adhesion plays a decisive role on overall properties of composites [18]. Thus, the interfacial shear strength (IFSS), as one of the most effective parameters for evaluating the efficiency of load transfer between fibers and matrix, has become a hot spot for the research field of fiber-reinforced composites. Several studies have been conducted with regard to IFSS for synthetic fiber composites by various characterization technologies. For example, Shin et al. [19] studied the interfacial properties between glass fibers and epoxy resin using the fiber fragmentation test, indicating that epoxy resin with a high curing agent content could promote interfacial wettability, resulting in superior material performance. Wang et al. [20] reported that the increase in contact area between glass fiber and ZnO nanowires led to an improvement of load transfer efficiency by micro-bond testing so as to enable the IFSS increase. Ren et al. [21] employed a single fiber pull-out test to determine the IFSS of bamboo fibers/PP; the results show that this value was dominated by several factors, such as fiber structure, embedded length, and fiber diameter. In addition, Le Duigou et al. [22] argued that there was a complete difference in fracture mechanisms for bamboo fiber composites with thermosetting and thermoplastic resins. Although many scholars have performed substantial investigations on the mechanical properties of bamboo fiber composites with chemical modification, very limited efforts have been endeavored to the interface bonding and its correlative mechanism. Evaluation of interfacial performance between epoxy resin and bamboo fibers is therefore of critical importance in the safe design of such composite structures.

In this work, the influence of fiber surface treatment on the performance of bamboo fiber reinforced epoxy-based composites was examined by experimental investigations in terms of interface characterization as well as thermo-mechanical properties. Scanning electron microscopy (SEM), differential scanning calorimetry (DSC), X-ray diffraction (XRD), thermogravimetric analysis (TG), tensile tests, and single fiber pull-out tests were conducted to observe changes on the composite properties before and after fiber treatment. This study is expected to provide detailed information to support the development and application of cost-effective and eco-friendly bio-composites, and to further explore the interfacial adhesion mechanism of thermoset composites reinforced with bamboo fibers from the perspective of micro-mechanics.

## 2. Results and Discussion

### 2.1. Mechanical Properties

Composite mechanical properties are provided in Table 1, and their significant differences are also shown. It was found that the composite tensile strength for 1%, 4%, and 7% is increased by 17.78%, 45.24%, and 28.92%, respectively, as compared with 0%. This is attributed to the fact that hemicellulose, pectin, and part of lignin of bamboo fibers are effectively removed by the NaOH solution [15], promoting the exposure of a large amount of micro-fibrils on the fiber surface to ensure favorable contact with the epoxy resin. Thus, interface adhesion improvement enhances the composite tensile performance [17]. However, it is noteworthy that NaOH at higher concentrations (7%) could destroy the hydrogen bond embedded in the micro-fibril and lead to fiber dissolution, resulting in fiber breakage [17]. Therefore, higher alkali concentration has a negative impact on the composite mechanical performance.

As shown in Table 1, there is a decreasing trend for the composite tensile modulus with increasing alkali concentration, indicating that while the interfacial bond is improved by alkali treatment, the modulus is characterized by a low deformation parameter being not sensitive to the adhesion [23]. In contrast, the conversion of cellulose I to cellulose II after fiber surface modification is the reason for a higher tensile modulus in untreated composites [24]. 

**Figure 1:**
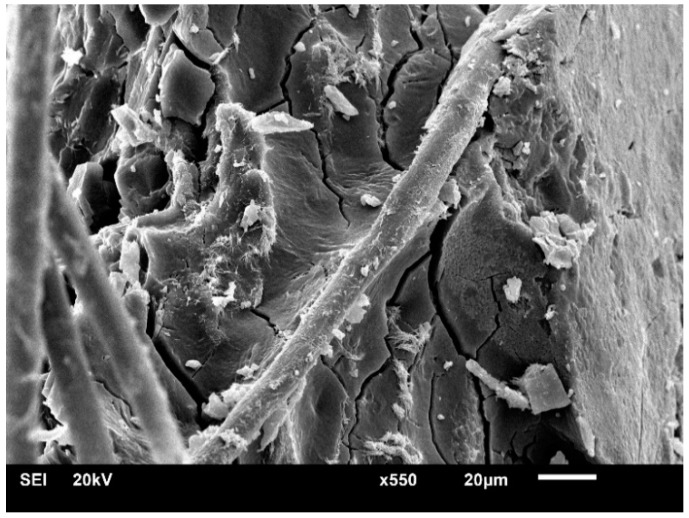
Interfacial morphology of epoxy-based composites.

It is well known that composite toughness/brittleness is dominated by the adhesion between fiber and matrix [23]. As is clearly observed in Table 1, the elongation at break of composites is found to increase from a concentration of 1% to 7%, which is increased by 15.92%, 23.32%, and 41.70%, respectively, when compared to 0%. Figure 1 represents the well-bonded interfacial morphology after the tensile test. Indeed, the alkali treatment results in a gradual removal of hemicellulose and lignin from raw bamboo fibers to refine celluloses with relatively high content. Consequently, the amount of hydroxyl groups increase significantly so that the hydrogen bonding in the network structure is disrupted, leading to disordered crosslinking of matrix molecular chains [12]. It can be found that the fiber hinders the matrix crack propagation, leading to larger elongation. As the case of alkali concentration for 7%, the elongation at break reaches the maximum, which is attributed to the fiber crystallinity decrement caused by the alkali treatment [17]. Thus, the amorphous region increases, causing an improved composite ductility [25]. According to the *p* value, the alkali treatment is found to have a significant influence on the composite properties, except for the case of tensile modulus at 1%.

### 2.2. Fracture Morphology

**Figure 2:**
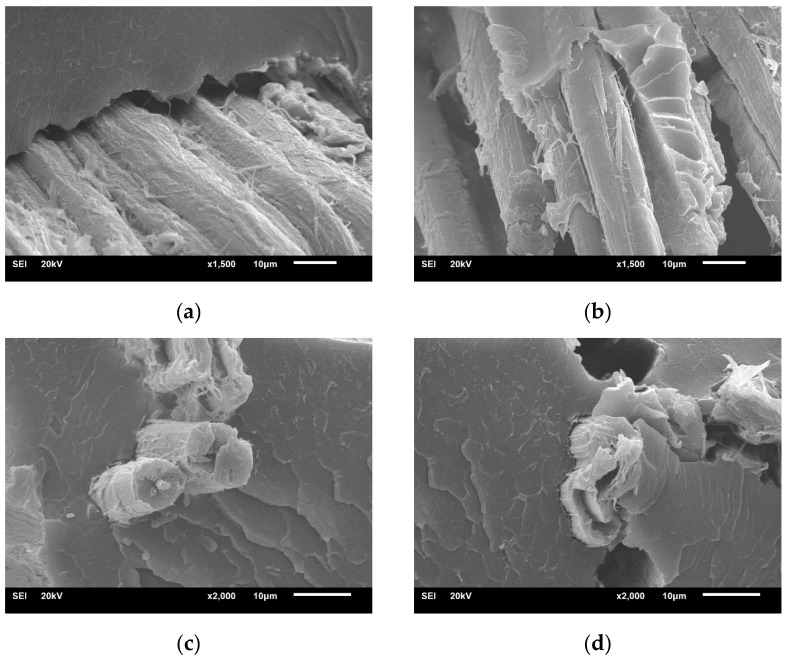
Interface fracture morphology of epoxy-based composites treated with various alkali concentrations of (**a**) 0%; (**b**) 1%; (**c**) 4%; and (**d**) 7%.

The SEM images of Figure 2 represent the composite fracture morphologies under various cases. As shown in Figure 2a, the fiber is completely pulled out from the matrix, as the evidence of a noticeable separation between elementary fibers and epoxy resin section. Interestingly, there is a tight connection among elementary fibers in fiber bundles, indicating that the presence of adhesive materials prevents the resin penetrating into elementary fibers. Thus, the interface compatibility becomes poor, leading to interfacial bonding deterioration.

It could be discovered from Figure 2b that 1% alkali concentration promotes the dispersion of elementary fibers, and then there is an increase in effective surface areas available for contact with the matrix. Thus, the resin infiltrates the gaps of cellulose fibers after alkali treatment [26], resulting in improved interface adhesion. Besides, the partial breakage of fibers appears in the composite. Figure 2c shows the fracture topography for composites treated by 4% alkali concentration. It is observed that broken fibers are tightly embedded in the epoxy resin when the composite failure occurs, indicating that the fiber bears considerable loads and are transferred to the matrix through the well-bonded interface. When the alkali concentration is 7%, it is also found that there is a well-bonding interface and some fiber pull-out in the matrix, as observed in Figure 2d. This phenomenon is explained by the fact that the fiber dissolution by 7% NaOH solution causes the emergence of considerable disordered microfibrils in the fiber surface, which is harmful to the interfacial properties [16]. In addition, there are more obvious wrinkles appearing on the cross-section of the resin after fiber treatment, leading to improved composite toughness. From the above-mentioned analysis, it can be concluded that composite mechanical properties are dominated by interfacial performance.

### 2.3. Interfacial Properties

**Figure 3:**
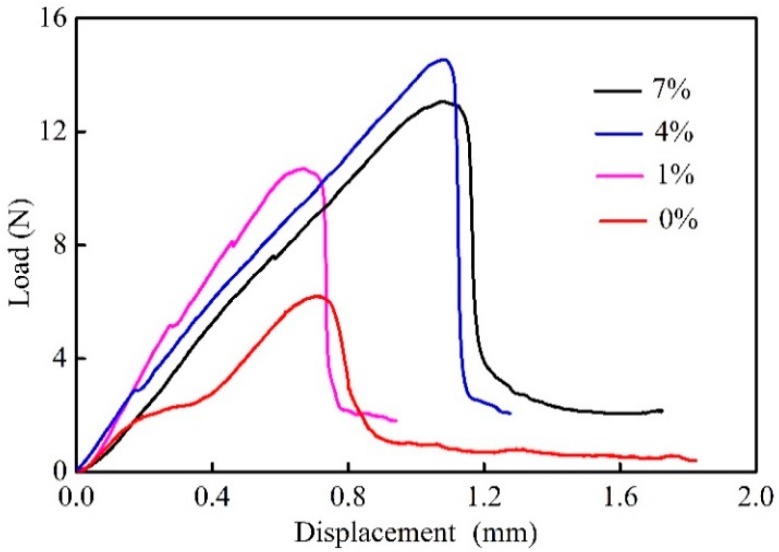
Interfacial morphology of epoxy-based composites.

Figure 3 illustrates several typical displacement-load curves obtained from micro-bond tests for fiber/matrix, and similarly they exhibit two characteristic loading-bear stages during the test.

In the initial phase, the force increases linearly with the displacement, and then the elastic energy accumulates continuously. When the load reaches the critical value, the interface is unable to withstand the applied force on the fiber, causing a debonding with the fiber apart from the resin. Consequently, the stored elastic energy is released in the creation of an interfacial crack, resulting in a sharp drop in load [27]. Before the fiber is completely pulled out from the resin, a periodic pressure is acted on the micro-droplet, due to the repeated intermittent contact between the clamp and the droplet. Thus, there is a dynamic friction force occurring between the fiber and the resin [27,28], causing a fluctuation in the load. Notably, a small drop is observed early in the loading curve of untreated composites, which is probably due to the occurrence of interfacial slipping between the fiber and resin [12]. This phenomenon can be explained by the poor wettability of resin and fiber in this case, thereby producing lower interfacial strength.

Table 2 shows that as compared with 0%, the IFSS increases by 30.79%, 100.30%, and 53.66% in the samples treated with 1, 4, and 7% NaOH solution, respectively, indicating that there is a maximum value at the 4% concentration. According to the previous work [15], a large number of elementary fibers split by NaOH are embedded in the matrix, and thus they bear part of the applied loads that are uniformly transmitted to the matrix, thereby improving the stress transfer efficiency. Wang et al. [17] argued that the presence of holes and gaps exposed on the fiber surface treated with 4% alkali concentration contributes to the formation of mechanical anchoring with the matrix. However, the cellulose crystal structure is damaged and the polarity is enhanced by the 7% concentration, leading to the IFSS decrease.

**Figure 4:**
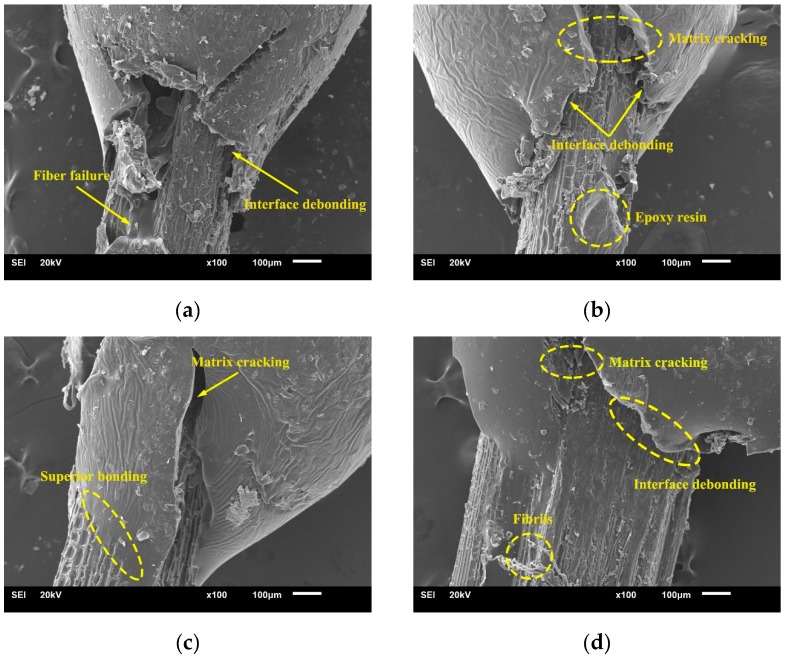
Interface morphology of epoxy-based composites treated with various alkali concentrations of (**a**) 0%; (**b**) 1%; (**c**) 4%; and (**d**) 7%.

Typical SEM images that show the interfacial morphology of fiber/matrix after the micro-droplet debonding test are presented in Figure 4a–d.

As observed from Figure 4a, the stress concentration caused by interfacial shear deformation accumulates around the fiber in the untreated sample. When the shear stress reaches the critical value of IFSS, the crack generated by interface debonding splits longitudinally along the fiber direction. This phenomenon proves that the interface adhesion is poor for the untreated fiber. Thus, there are two failure modes, namely, fiber/matrix debonding and fiber breakage. In Figure 4b, the droplet is found to be cracked and the fiber surface is attached to the broken droplet, which could be attributed to the fact that matrix crack propagates along the weak interface, resulting in the debonding of fiber from the matrix. Thus, interface and matrix failures appear in the case of 1% alkali-treated fiber, suggesting that interfacial adhesion improved after the treatment. Such a case can be also observed in Figure 4c, indicating that the fiber/matrix interface is partially debonded and the matrix is cracked. This phenomenon suggests that the 4% alkali treatment has a more positive effect on fiber/matrix incorporation. Figure 4d shows that many fibrils are detached mutually in the fiber sample, indicating the occurrence of fibrillation when the alkali concentration is 7%. This could be explained by the fact that the fiber internal structure is damaged with the help of higher concentration, and consequently the fibril division appears prior to interface failure. Accordingly, the sample is characterized by the fiber and matrix failures. The above-mentioned analysis concluded that stronger interfacial fiber-matrix adhesion is achieved through the fiber surface modification.

### 2.4. Glass Transition Temperature

**Figure 5:**
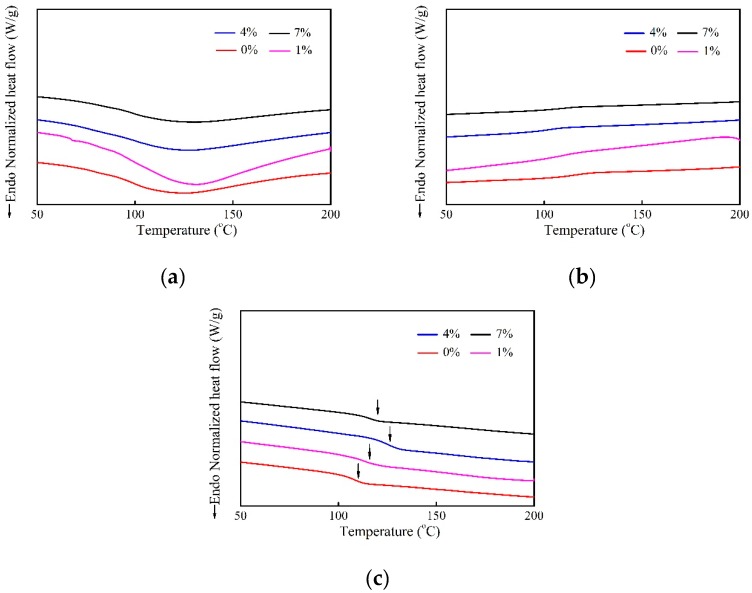
DSC curves of epoxy-based composites treated with various alkali concentrations for: (**a**) the first heating; (**b**) the cooling; and (**c**) the second heating process.

DCS curves of untreated- and treated-fiber composites with various alkali concentrations are shown in Figure 5. It is noteworthy that the glass transition temperature (*T*_g_) is marked with an arrow, as observed in Figure 5c. In Figure 5a, since the thermal behavior during the heating process is affected by several factors such as moisture and induced internal stress [29], the samples are first heated to 200 °C to eliminate the influence of the previous thermo-mechanical history. Interestingly, there is no crystallization exotherm of composite appearing in Figure 5b, indicating that the epoxy-based composites did not crystallize during the first cooling process. Also, Figure 5c shows that there is no significant exothermic peak during the second heating run, suggesting that the curing reaction is nearly complete. 

Table 3 exhibits *T*_g_ of the composites treated with various alkali concentrations. It was discovered that when the concentration is 1%, 4%, and 7%, respectively, *T*_g_ is increased by 2.97%, 12.96%, and 6.76%, respectively, as compared with the untreated composite. In fact, the interface compatibility of the fiber/matrix is weak in the untreated samples, promoting high mobility of epoxy molecular chains along the interface region. In contrast, the alkali treatment makes those molecular chains more constrained by being anchored to the immobile crystallites [30,31]. Therefore, the mobility of resin molecular chain closest to the fiber is degraded, resulting in higher *T*_g_.

### 2.5. Thermal Stability

**Figure 6:**
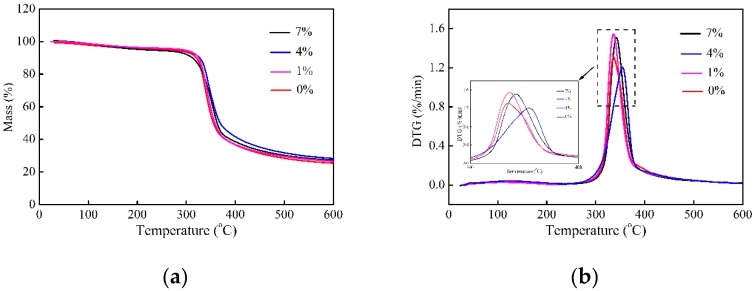
(**a**) TG; and (**b**) DTG curves of bamboo fibers and epoxy-based composites treated with various alkali concentrations.

Figure 6 presents TG and its derivative thermograms (DTG) curves of epoxy-based composites. The peak temperatures and the weight loss at 600 °C obtained from DTG curves are summarized in Table 4. 

It is seen from the TG curve that the main degradation stage is in the range of 270 °C and 400 °C. Herein, bamboo fibers and epoxy resin are decomposed substantially. The weight loss is attributed to the decomposition of aromatic groups and aliphatic amine curing agent of epoxy network, as well as the depolymerization and degradation of hemicellulose and cellulose [32,33]. When the temperature reaches 600 °C, the residue belongs to amorphous carbon. From Table 4, the decomposition temperature with 1%, 4%, and 7% is found to be increased by 1.17%, 2.95%, and 1.31%, respectively, as compared with the sample of 0%. This suggests that the interface bonding needs to be destructed prior to fiber and matrix decompositions, which is due to superior interfacial adhesion caused by the alkali treatment. Thus, the thermal stability of alkali treated composites is improved availably. In addition, the weight loss at 600 °C for the composites of 1%, 4%, and 7% is decreased by 1.55%, 3.77%, and 3.00%, respectively, as compared with 0%, indicating that there is more amorphous carbon remaining in the alkali-treated composites. It is explained by the fact that the amorphous carbon acts as a protective layer to reduce the penetration of volatile degradation products into composites and prevent further degradation, producing higher heat resistance [28].

## 3. Materials and Methods 

### 3.1. Materials

Bamboo fibers used in this work were extracted from a six-year-old Moso bamboo produced in Fujian Province, China, by using the blasting extraction method. The bamboo fiber density was 1.035 g/cm^3^, and the fiber diameters were found to range from 200 μm to 600 μm [34]. Epoxy resin (brand YJ-80) as the matrix was supplied by Wells Advanced Materials Co., Ltd. (Shanghai, China), in a viscous state at room temperature. Thermo-mechanical characteristics of the resin sample were provided by the supplier, as listed in Table 5. The curing agent was polyamide resin (brand SF-650-200) with an amine value of 220 ± 10 mgKOH/g and viscosity at 40 °C of 15000–25000 mPa·s, delivered from Shanfeng Chemical, Ltd. (Changzhou, China).

### 3.2. Alkali Treatment

Based on our previous work [15,16], bamboo fibers were soaked in various NaOH concentrations of 1, 4, and 7% for 3 h at room temperature. Afterwards, the treated fibers were taken out from the solution and washed with distilled water for 3–4 times to remove the residual solution from the fiber surface. The washed fibers were dried in an oven at 80 °C for 8 h to reduce moisture content and then stored in a desiccator (ZKF040, Shanghai Experimental Instrument Factory Co., Ltd., Shanghai, China) with sealed polyethylene bags.

### 3.3. Composite Preparation

All the composites of fiber volume fraction of 50% (*V*_f_ = 50%) were manufactured by hot-pressing preforms with a press-molding machine (Y/TD71-45A, Tianduan Press Co., Ltd., Tianjin, China) [35]. The mold size for the composite plate was 175 × 170 × 2 mm^3^, and inner surfaces of upper and lower plates were coated with a polystyrene stripper (Dongheng Chemical Industry Co., Ltd., Shanghai, China). Prior to composite fabrication, the bamboo fibers were uniformly mixed with epoxy resin in the mold. They were dried in an oven at 80 °C for 2 h to remove all absorbed and adsorbed moisture and to eliminate voids. The vulcanization moulding machine (XLB-0050, Jinrunqi, Qingdao, China) was preheated at 80 °C without pressure for 10 min to promote the penetration of resin into fiber, and hot pressing was conducted on the samples for 5 h at 100 °C. Afterwards, the samples were cured at room temperature for 11 h and maintained in the moulding machine at 80 °C for 8 h. After these steps, the composite plate was removed and cut into the required dimensions of 150 × 20 × 2 mm^3^ using a dedicated cutting machine with a diamond-coated cutting blade. Bracing cleats made of aluminium were glued to each end of the samples. It should be emphasized that configuration was limited to unidirectional lamina with a ply angle of 0° in this work. Evidently, the length of continuous bamboo fiber had to be equal to that of the specimen, namely, 150 mm. Thus, fibers with an average length of 170 mm were used through artificial selection before composite preparation to guarantee length consistency. In addition, the fibers were visually selected to verify the absence of defects on the surface, because they were plant-based fibers with geometric irregularities. For the purpose of simplicity, the abbreviations of ‘0%’, ‘1%’, ‘4%’, and ‘7%’ represent composites with bamboo fibers treated with alkali concentrations of 0%, 1%, 4%, and 7%, respectively.

### 3.4. Composite Characterization

#### 3.4.1. Tensile Test

The tensile strength, modulus, and elongation at break of composite samples were measured using an electromechanical universal testing machine (CMT4204, Skyan Power Equipment Co., Ltd., Shenzhen, China) fitted with a Celtron 50 kN load cell (model PSD-5tSJTH, serial number 33766) in accordance with ASTM D638-10 [36]. Five samples of each composite were loaded on a displacement control system with a cross-head speed of 5 mm/min at room temperature under 50% humidity. Afterwards, the average values and standard deviations were recorded for the following analysis.

#### 3.4.2. Micro-Bond Test

**Figure 7:**
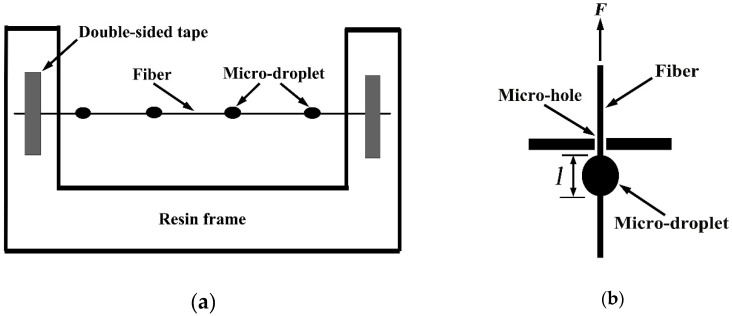
Schematic of (**a**) specimen preparation and (**b**) micro-bond test.

The sample preparation for the micro-bond test is shown in Figure 7. The epoxy resin liquid was uniformly mixed with epoxy resin and a curing agent (Polyamide) at the weight ratio of 1:1, and placed in a vacuum oven for 15 minutes to remove air bubbles on the liquid surface as far as possible. Then, the fiber was fixed and straightened between two paper frames, and was dripped onto the epoxy resin droplet with 2 mm diameter by a needle tip. After curing at room temperature for 24 h, the samples were stored in sealed polyethylene bags.

**Figure 8:**
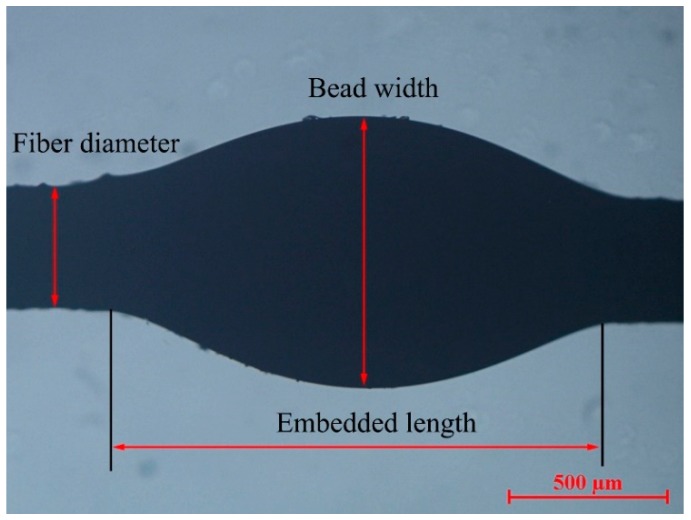
Morphological image of the micro-droplet sample.

Single fiber testing machine (Donghua University, Shanghai, China) was employed to measure the IFSS of samples by the micro-bond test, the schematic diagram of which is shown in Figure 7. After the droplet was fixed by the micro-pore, an external load was applied along the axial direction till the droplet was debonded from the fiber. The critical load was recorded, and consequently the IFSS was determined from the following mechanical formula (Equation (1)) when the fiber diameter and micro-droplet length were known. The loading rate of chuck was 2 mm/min and sample clamping length was 20 mm.

(1) $$\mathrm{IFSS}=\frac{F}{\pi dl}$$

where *F* is the critical load. *d* and *l* denote the fiber diameter and the droplet length, respectively. The fiber and droplet morphologies were photographed using a professional polarizing microscope (LV100 POL, Nikon, Tokyo, Japan). The fiber diameter and droplet length were measured using a self-contained image analysis tool, as shown in Figure 8.

#### 3.4.3. Scanning Electron Microscope

The morphological characterization for fracture surface and fiber/resin morphology after the tests were examined by SEM (JELO JMS-6610, Tokyo, Japan) in high vacuum mode at an accelerating voltage of 10 kV. For SEM observations, all the samples were sputtered with a thin layer of gold using an ion sputter coater (SBC-12, KYKY, Beijing, China) for approximately 90 s to prevent charging.

#### 3.4.4. Differential Scanning Calorimeter Analysis

Glass transition behavior was evaluated by DSC Q20 analyzer (TA Instruments, New Castle, DE, USA) under a nitrogen atmosphere (80 mL/min); about 3–5 mg samples were tested according to the following three steps: heating from 30 °C to 200 °C and held for 5 min to remove the effect of thermal and mechanical histories, then cooling to 30 °C and kept for an additional 5 min. At the end, the samples were heated to 200 °C. All the above processes were performed at a rate of 10 °C/min.

#### 3.4.5. Thermogravimetric Analysis

The thermal stability of fiber, epoxy resin, and composites were tested by TG using a synchronous differential thermal analyzer (STA409PC, NETZSCH, Selb, Germany) under nitrogen atmosphere with a flow of 50 mL/min. About 3–5 mg samples were operated in a continuous heating mode from 200 °C to 600 °C at a heating rate of 10 °C/min. The weight loss of various samples at 600 °C was determined by % weight loss using the following equation:

Weight loss (%) = (*W*_1_ − *W*_2_)/*W*_1_ × 100, (2)

where *W*_1_ is the sample weight prior to test and *W*_2_ is the one at any given temperature.

## 4. Conclusions

In this work, bamboo fibers are treated with alkali treatment for 1%, 4%, and 7% concentrations and the ensuing epoxy-based composites are prepared by hot molding technology. The effects of alkali concentrations on thermal-mechanical and interfacial properties of bamboo fiber/epoxy composites are investigated through experimental measurements. The conclusions are outlined as follows:

(1) The composite tensile strength is improved as the alkali concentration increases. However, when the concentration reaches 7%, the micro-fibril structure of the bamboo fibers is damaged, and contrarily the interfacial adhesion becomes weakened. Thus, the maximum value in composite strength is at 4% concentration. In this case, the interface compatibility also becomes optimal, as proved by the fracture morphology. Besides, the elongation at break increases as the concentration increases.
(2) The IFSS of the fiber/matrix increases with increasing alkali concentrations, and reaches maximum value at a concentration of 4%. This alkali concentration strengthens the mechanical interlocking of the fiber surface with epoxy resin, enhancing the interface bonding. Thus, the interface failure is mainly caused by matrix damage.
(3) The mobility of the epoxy molecular chains is restricted after the alkali treatment, leading to higher *T*_g_. It is concluded that the alkali treatment could improve decomposition temperature and increase the residual mass, exhibiting improved thermal stability.

## Figures and Tables

**Table 1:** Mechanical properties of composites treated with various alkali concentrations.

Samples	Tensile Strength (MPa)	*p* (α = 0.05)	SD	Tensile Modulus (GPa)	*p* (α = 0.05)	SD	Elongation at Break (%)	*p* (α = 0.05)	SD
0%	134.32 ± 19.77	-	-	0.82 ± 0.13	-	-	4.46 ± 0.10	-	-
1%	158.20 ± 11.63	0.048	Yes	0.77 ± 0.05	0.268	No	5.17 ± 0.19	0.027	Yes
4%	195.09 ± 17.91	0.007	Yes	0.72 ± 0.02	0.031	Yes	5.50 ± 0.32	0.002	Yes
7%	173.17 ± 19.38	0.031	Yes	0.66 ± 0.04	0.012	Yes	6.32 ± 0.33	0.002	Yes

Note: Two-sided *t*-test in ANOVA, and *α* is the significant level. For *p* ≥ 0.05, there is no significant difference (SD).

**Table 2:** IFSS for the epoxy resin with bamboo fibers treated with various alkali concentrations.

Samples	IFSS (MPa)	*p* (α = 0.05)	SD
0%	3.28 ± 0.34	-	-
1%	4.29 ± 0.56	0.009	Yes
4%	6.57 ± 0.69	0.0001	Yes
7%	5.04 ± 0.32	0.00002	Yes

Note: Two-sided *t*-test in ANOVA, and *α* is the significant level. For *p* ≥ 0.05, there is no significant difference (SD).

**Table 3:** Glass transition temperature of epoxy-based composites treated with various alkali concentrations.

Samples	*T*_g_ (°C)	*p* (α = 0.05)	SD
0%	109.37 ± 0.87	-	-
1%	112.62 ± 1.24	0.021	Yes
4%	123.54 ± 2.22	0.0005	Yes
7%	116.76 ± 0.81	0.0004	Yes

Note: Two-sided *t*-test in ANOVA, and *α* is the significant level. For *p* ≥ 0.05, there is no significant difference (SD).

**Table 4:** Comparison of peak temperature and weight loss of epoxy-based composites treated with various alkali concentrations.

Samples	Peak Temperature (°C)	*p* (α = 0.05)	SD	Loss Weight at 600 °C (%)	*p* (α = 0.05)	SD
0%	342.4 ± 1.50	-	-	73.42 ± 1.28	-	-
1%	346.4 ± 1.27	0.023	Yes	72.28 ± 0.33	0.015	Yes
4%	352.5 ± 1.08	0.0002	Yes	70.65 ± 1.26	0.012	Yes
7%	346.9 ± 1.54	0.011	Yes	71.22 ± 1.06	0.015	Yes

Note: Two-sided *t*-test in ANOVA, and *α* is the significant level. For *p* ≥ 0.05, there is no significant difference (SD).

**Table 5:** Thermo-mechanical properties of epoxy resin sample.

Epoxide Equivalent Weight (g/mol)	Viscosity (mPa·s at 70 °C)	Solubility	Glass Transition Temperature (°C)	Tensile Strength (MPa)	Tensile Modulus (MPa)	Elongation at Break (%)
180–195	600–1000	Insoluble in water	≥150	21	480	3.8
